# Editorial: NAD+ metabolism as a novel target against infection–Volume II

**DOI:** 10.3389/fmolb.2022.1087897

**Published:** 2022-11-21

**Authors:** Enrico Balducci, Nady Braidy, Marie Migaud

**Affiliations:** ^1^ School of Biosciences and Medicine Veterinary, University of Camerino, Camerino (MC), Italy; ^2^ Centre for Healthy Brain Ageing, School of Psychiatry, University of New South Wales, Sydney, NSW, Australia; ^3^ Department of Pharmacology, Mitchell Cancer Institute, F. P. Whiddon College of Medicine, University of South Alabama, Mobile, AL, United States

**Keywords:** antibiotic resistance, NAD metabolism, bacteria, immune response, infectious diseases

Fundamental discoveries in the history of science, including research from four Nobel laureates, have identified the pyrimidine nucleotide, nicotinamide adenine dinucleotide (NAD+) as an essential metabolite and the most important cross-kingdom electron carrier. While a wealth of scientific studies in the last century have highlighted the important role of NAD+ in cellular energy generation and redox biology, recent studies have provided evidence for the paramount importance of the non-redox functions of NAD+. Alteration in intracellular NAD+ concentrations is critical in several pathological conditions, including but not limited to neurodegenerative diseases, cancer, diabetes, ischemia injury, dysmetabolic diseases, and inflammatory disorders. Recent advances in our understanding of the biological functions of NAD+-consuming enzymes and the role of NAD+ biosynthetic routes have shown that targeting NAD+ metabolism has very promising potential for therapeutic treatment of infectious diseases. Bacterial resistance to antibiotics is considered one of the greatest threats to human health. Cases of antibiotic resistance are constantly emerging, and the time needed for bacteria to become resistant to newly introduced antibiotics, is getting shorter. Therefore, it is crucial to identify antimicrobials with new mechanism of action to use as therapeutic tools. In this context, modulation of host NAD+ metabolism by the activation or inhibition of key enzymes could influence NAD+ signaling pathways in pathogens and their ability to colonize host cells. Further, the discovery of the enzymes involved in NAD+ turnover in the host during infection could increase our knowledge of bacterial pathogenesis and may be relevant for the development of targeted drug therapies against antibiotic resistant bacteria. However, detailed mechanisms by which NAD+ acts as a regulator and how we can target NAD+ metabolism during microbial infections is still poorly understood.

In the current Research Topic, the published papers collectively help to gain some cardinal knowledge on the functions of NAD+ and precursors during infection, and cancer and to find new potential therapeutic targets.

One original paper described the role of Nicotinamide Mononucleotide (β-NMN), a key precursor molecule in the NAD salvage pathway, in reducing inflammation in bacterial peritonitis. Other NAD precursors such as Nicotinamide Riboside (NR) or Nicotinamide have been shown to reduce oxidative stress and inflammation in the heart or to increase the survival rate of mice with lethal endotoxemia or experimentally induced polymicrobial sepsis, respectively. More recently β-NMN administration was effective in reducing the pro-inflammatory cytokine production in an LPS-induced inflammation model. Combining transcriptome analysis and biological assays, Cros et al. demonstrated unequivocally and in agreement with existing data, the advantage to treat bacterial infections with β-NMN compared to other molecules affecting NAD+ metabolism. They also showed that the reduced dysregulated inflammation after the treatment with β-NMN is correlated to reprogramming of macrophages toward an anti-inflammatory pathway. Overall, the promising data presented by Cros et al., confirm the potential therapeutic use of β-NMN in the treatment of bacterial infections and in reducing their deleterious effects.

In the second paper Baquero et al. carefully reviewed and analyzed the correlation between NAD+ metabolism and the antimicrobial effects exerted by the intestinal microbiota. The administration of NAD+ precursors synergize with intestinal microbial populations to enhance NAD+ levels in host mammalian cells suggesting that a healthy gut microbiota is strictly necessary for the synthesis of key components used in alternative NAD+ biosynthetic pathways in the host. Baquero et al. highlighted the importance of the microbiota composition and activity when drugs interfering with NAD+ metabolism are used and developed. The cross talk between host and microbiota has been shown in many experimental conditions and should be considered when targeting NAD+ homeostasis in the host. As pointed out by Baquero et al., a correct use of NAD+-boosting compounds accelerating the bacterial metabolism can reduce the persistence of antibiotic resistant pathogens. Finally, they demonstrated that NAD+ availability can influence the growth of bacterial populations producing low-molecular weight antimicrobial agents such as microcin able to modulate the host microbiome.

Among the NAD+ consuming enzymes, CD38 plays a key role in prostate cancer cells since its expression is downregulated if compared to normal tissues. However, a clear role of CD38 in enhancing tumor progression is still lacking. Analyzing oxygen consumption, intracellular NAD+ levels and cell viability Kanayama and Luo demonstrated the close correlation of CD38 overexpression and cancer phenotypes in a stable prostate cancer cell clone. They also observed that while the NAM supplementation failed to restore normal NAD+ levels, it rescued CD38 induced apoptosis and mitochondrial stress. However, they claimed that additional studies are required to confirm the therapeutical implications for NAM treatment in prostate cancer.

Although Isocitrate Dehydrogenase (IDH) is a well-known metabolic enzyme playing a key role in the citric acid cycle, studies have recently evidenced that after mutations in key arginine residues IDH acquires a novel function for which NADP+ instead of NAD+ is the preferred cofactor. Under such circumstances, IDH catalyzes the synthesis of hydroxyglutarate, an oncometabolite that promotes tumorigenesis *via* the reprogramming of DNA and histone methylation. These observations demonstrate that IDHs are not only key metabolic enzymes but are also important clinical diagnostic biomarkers for certain tumors as well as potential targets for therapeutic drugs. Huang et al. performed a detailed biochemical characterization of a novel NADP+-specific type III IDH from the eukaryotic microalga *P. tricornutum*. Among the biochemical properties studied, of interest is the identification of key residues, pivotal for coenzyme binding and specificity. While the IDH studied is derived from a microalga, the data shown provides a better understanding of the catalytic and regulatory properties of this family of enzymes as it affects the metabolism of NAD+ and its functions.

NAD+ metabolism is a highly interdisciplinary research field with many open questions still to be addressed to improve our knowledge into the various implications of its metabolism and regulatory functions in biological processes. In conclusion, this Research Topic provides examples of how the application of different approaches can benefit the scientific community by providing new insights into the many aspects of NAD+ metabolism.

